# Coinheritance of the c.-19 G > C and c.315 + 1 G > A Variants in the *β*-Globin Gene Leads to Thalassemia Disease: A Report from the North of Iran

**DOI:** 10.1155/2023/9950421

**Published:** 2023-08-28

**Authors:** Hossein Jalali, Mahan Mahdavi, Mohammad Eslamijouybari, Mohammad Reza Mahdavi

**Affiliations:** ^1^Thalassemia Research Center, Hemoglobinopathies Institute, Mazandaran University of Medical Sciences, Sari, Iran; ^2^Sinaye Mehr Research Center, Sari, Iran; ^3^Gastrointestinal Cancer Research Center, Mazandaran University of Medical Sciences, Sari, Iran

## Abstract

Up to now, more than 300 pathogenic variants have been identified in the *β*-globin gene, some of which are categorized as silent mutations that do not change the hematological indices. In the present study, our aim is to introduce the first report of a case with thalassemia intermedia with coinheritance of the c.315 + 1 G > A pathogenic variant and a silent variant (HBB: c.-19 G > C) that was missed during the screening program. Multiplex-Gap-PCR and Sanger sequencing methods were applied to identify *α-* and *β*-globin gene mutations in a 26-year-old male subject with diagnosis of thalassemia. The identified mutations were also checked on the parent's sample. The CBC and capillary electrophoresis tests were performed on the parent's blood samples. The case was compound heterozygote for the c.315 + 1 G > A and c.-19 G > C (rs1239893012) variants. The subject's mother carried the c.-19 G > C variant in the *β*-globin gene while her CBC and electrophoresis test results showed a normal pattern. Silent mutations are susceptible to being missed during premarital screening of *β*-thalassemia carriers, and the c.-19 G > C variant is recommended to be classified as a pathogenic variant in the *β*-globin gene.

## 1. Introduction

*β*-Thalassemia is one of the most frequent inherited diseases caused by defects in the synthesis of hemoglobin's *β* subunit [1, 2]. The *HBB* gene is located on the short arm of chromosome 11, with a size of 1.6 kb, and consists of three exons. Of the 900 genomic alterations in the *β*-globin, over 300 variants result in *β*-thalassemia worldwide [2]. According to the amount of *β*-globin production, *β*-globin mutations have been classified into *β*^+^ and *β*^0^. *β*^0^ mutations completely inactivate the *β*-globin gene, leading to no *β*-globin chain production, while other mutations allow the production of some *β*-globin subunits [3].

*β*-Thalassemia is the most common form of transfusion-dependent thalassemia in Iran, especially in the south and north regions with a carrier frequency of more than 10% [4–6]. Since the start of premarital carrier screening programs in 1991, a significant reduction has been observed in the emergence of new cases in most regions of the country [7]. However, there are still some problems in controlling the birth of new subjects [8]. Based on the screening process, marriage registrars refer all couples to a designated local laboratory for premarital screening. In the lab, the first man's red cell indices are checked, and if microcytosis (mean cell hemoglobin <27 pg or mean red cell volume <80 fl) is observed, the woman is also tested. When both are microcytic, their HbA2 concentrations are measured, and if both have HbA2 concentration above 3.5%, they are referred for genetic counseling [7]. The carriers of *β*-thalassemia usually show low hematological indices that are accompanied by higher levels of HbA2 (≥3.5%). However, there are some silent mutations on *β*-globin that does not change the HbA2 level and may lead to misdiagnosis of the carriers [9, 10].

At the present study, we aimed to present the first report of a case with thalassemia intermedia who coinherited the c.315 + 1 G > A pathogenic variant and a novel variant (HBB: c.-19 G > C) that was missed during the screening program.

## 2. Materials and Methods

A 26-year-old male subject with the diagnosis of thalassemia based on hematological indices was referred to the Fajr Medical Genetics and Pathobiology Lab in Sari, Iran, for molecular investigation of thalassemia (Table 1). His hemoglobin level was 6 g/dl, and due to the low hemoglobin level, he had experienced irregular blood transfusions. During the premarital screening program for thalassemia, his parents were classified as a couple with no risk of having a child with thalassemia.

After obtaining written informed consent, molecular analysis was conducted on genomic DNA extracted from peripheral blood using QIAamp DNA Mini Kit (Qiagen, Germany). Multiplex Gap-PCR was performed to identify common Mediterranean *α*-globin gene deletions (-*α*^3.7^, -*α*^4.2^, --^MED^, and --^20.5^), and Sanger DNA sequencing method (3130XL, ABI, USA) was used to detect other mutations on *α-* and *β*-globin genes. The presence of the detected mutations was investigated in the parent's samples. CBC and capillary electrophoresis (Sebia, France) were also applied on the parent's blood sample.

## 3. Results

The results of the primary CBC test of the case were compatible with thalassemia disease (Table 1). The multiplex Gap-PCR results indicated that the case does not carry the common *α*-globin gene deletions. The Sanger sequencing results of the *α*-globin also showed no mutation.

**Figure 1:**
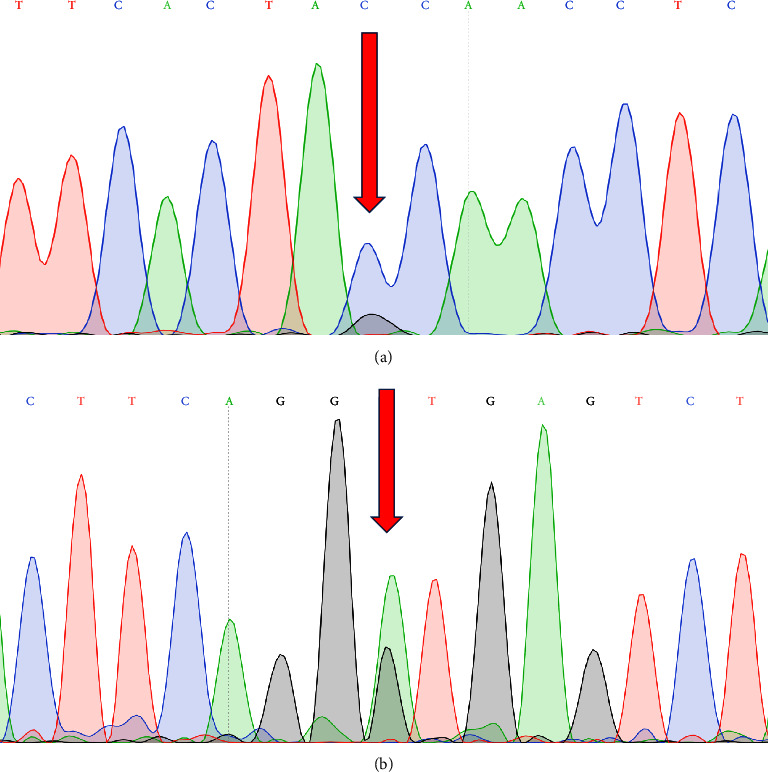
The results of Sanger sequencing test showed the presence of c.-19 G > C (a) and c.315 + 1 G > A (b) variants on the *β*-globin gene of the case.

The sequencing of *β*-globin gene revealed that the case is a compound heterozygote for the c.315 + 1 G > A and c.-19 G > C (rs1239893012) variants (Figure 1). The presence of the identified variants in the subject's parents indicated that her father and mother are heterozygote for the c.315 + 1 G > A and c.-19 G > C variants on the *β*-globin gene, respectively.

**Figure 2:**
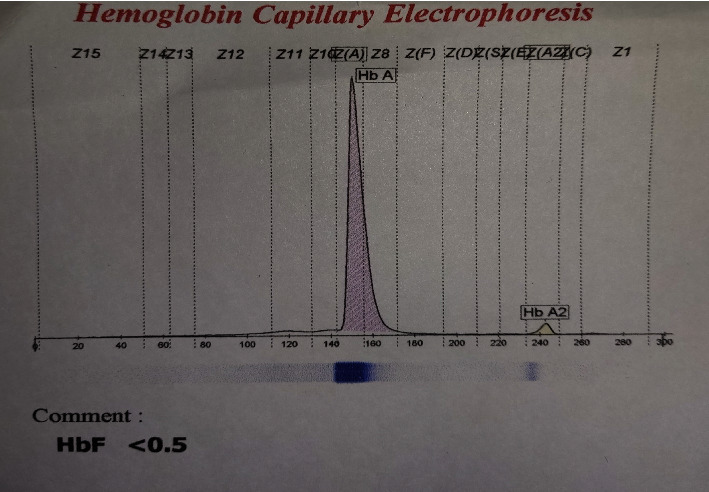
The results of capillary electrophoresis on the mother's blood showed a normal pattern, despite having c.-19 G > C variant. The mother has a normal HbA2 level, even though she carries the c.-19 G > C mutation.

The CBC and capillary electrophoresis test results of the father were compatible with a *β*-thalassemia carrier constitution. However, in the mother, a normal pattern was observed despite being heterozygous for the c.-19 G > C variant (Table 2 and Figure 2).

## 4. Discussion

CBC and MCV are the primary laboratory tests and the key indices for screening thalassemia carriers [11, 12]. The presence of microcytosis on CBC is the first indicator suggestive of thalassemia carriers. Other parameters used for carrier screening include Hb (normal/slightly decreased), mean corpuscular hemoglobin (MCH) (decreased), RBC (increased), and red cell distribution width (RDW) (normal) [12]. It should be mentioned that iron deficiency can also cause microcytic anemia, and a serum ferritin test can help differentiate it from other forms of anemia [13]. According to the mentioned criteria, carriers of silent mutations in the *β*-globin gene with normal CBC values may be missed because subsequent genetic investigation for thalassemia is not ordered in such individuals. In the national premarital screening program for thalassemia, if one case has normal hematological indices, the couple would be considered as a couple with no risk of having a child with thalassemia. Hence, based on screening program criteria and normal hematological indices of the mother, the parents of the presented case were considered as a couple who do not have the risk of childbirth with thalassemia and were ruled out of further investigations.

## Figures and Tables

**Table 1:** The hematological indices of the presented case with *β*-thalassemia.

The studied parameter	RBC^1^ (×10^6^/*µ*l)	Hb^2^ (g/dL)	MCV^3^ (fl)	MCH^4^ (pg)	M.C.H.C^5^ (g/dl)	R.D.W^6^ (micg/r)	Ferritin (ng/ml)
The case	2.86	6	74.5	21	28.2	33.6	906
Normal range	4.5–5.9	12–17	80–100	27−3	31–37	115−15	30–400

^1^Red blood cell. ^2^Hemoglobin. ^3^Mean corpuscular volume. ^4^Mean corpuscular hemoglobin. ^5^Mean corpuscular hemoglobin concentration. ^6^Red blood cell distribution width.

**Table 2:** Hematological indices of the parents of the case with thalassemia (the mother is a silent carrier).

	Variant of *β*-globin	RBC (×10^6^/*µ*l)	Hb (g/dL)	MCV (fl)	MCH (pg)	Hb-A (%)	Hb-A2 (%)	Hb-F (%)
Father	c.350 + 1 G > A	5.37	10	61.8	18.6	94.5	5.1	>0.5
Mother	c.-19 G > C	3.87	10.4	82.4	26.9	97.1	2.9	<0.5

## Data Availability

The data used in this study are available from the author upon request.
